# Key patient-reported outcomes in children and adolescents with intoxication-type inborn errors of metabolism: an international Delphi-based consensus

**DOI:** 10.1186/s13023-022-02183-2

**Published:** 2022-01-29

**Authors:** Florin Bösch, Nina A. Zeltner, Matthias R. Baumgartner, Martina Huemer, Markus A. Landolt

**Affiliations:** 1 Division of Metabolism and Children’s Research Center, University Children’s Hospital Zurich, University of Zurich, Steinwiesstrasse 75, 8032 Zurich, Switzerland; 2 Department of Psychosomatics and Psychiatry, and Children’s Research Center, University Children’s Hospital Zurich, University of Zurich, Zurich, Switzerland; 3 Division of Child and Adolescent Health Psychology, Department of Psychology, University of Zurich, Zurich, Switzerland; 4 Department of Paediatrics, LKH Bregenz, Bregenz, Austria

**Keywords:** Patient-reported outcomes, PROs, Patient-reported outcome measures, PROMs, Inborn errors of metabolism, Rare metabolic diseases, Organic acidurias, Urea cycle disorders, Maple syrup urine disease, Phenylketonuria

## Abstract

**Background:**

Acute intoxication-type inborn errors of metabolism (IT-IEM) such as urea cycle disorders and non-acute IT-IEM such as phenylketonuria (PKU) and their treatment have a major impact on the life of affected children and families. Yet patients’ and parents’ perspectives on the burdens of IT-IEM and its effects on everyday functioning and well-being have rarely been addressed. Patient- and observer-reported outcomes (PROs/ObsROs) are critically important to evaluate and target health care and treatment efficacy. Therefore, it is mandatory to define PROs/ObsROs relevant to patients with IT-IEM, their families, and health care professionals and to provide valid, standardised and reliable measuring instruments. To achieve consensus we performed a two-round, electronic-based modification of a Delphi survey including 27 parents of affected children, nine teenage patients and 35 health professionals (physicians, nutritionists, psychologists). The final set of PROs/ObsROs was discussed and defined in an online consensus meeting with a subsample of three health professionals, three parents and two patients. For this final set, appropriate measures (PROMs/ObsROMs) were assembled.

**Results:**

Seventeen PROs/ObsROs constitute the final core set for paediatric IT-IEM. They cover social (e.g. social participation), emotional (e.g. positive affect), and disease-related aspects (e.g. attitude towards treatment) of patients’ lives as well as the experience of parents (e.g. parental stress).

**Conclusion:**

To promote a holistic treatment approach, this consensus-driven set of relevant PROs/ObsROs should be incorporated into daily IT-IEM care and considered as the key psychological outcomes in clinical trials. We have identified existing—psychometrically and contextual—appropriate PROMs/ObsROMs with open access to facilitate this process.

**Supplementary Information:**

The online version contains supplementary material available at 10.1186/s13023-022-02183-2.

## Introduction

Intoxication-type inborn errors of metabolism (IT-IEM) are a group of rare, chronic diseases. Some take an acute course with metabolic crises (e.g. urea cycle disorders or organic acidurias); others, like phenylketonuria (PKU) are non-acute IT-IEM. In some disorders, even lifelong adherence to treatment such as protein-restricted diet, supplementation of amino acids, and medication may not prevent patients from neurocognitive impairment. Many patients experience variable somatic symptoms such as nausea or fatigue. Especially in children and adolescents the considerable burden of both disease and treatment may impair health-related quality of life (HrQoL) and result in emotional constraints [1, 2].

A patient-reported outcome (PRO) is a report about the subjective perception in relation to a health condition directly from the affected patient, without interpretation by physicians or others [3]. In paediatrics, an observer-reported outcome (ObsRO) is applied to either substitute or complement a PRO with the perspective of a proxy (primarily parents) [4, 5]. PROs and ObsROs are predominantly measured using questionnaires, so called patient- and observer-reported outcome measures (PROMs/ObsROMs). PROs (and ObsROs; applicable for the whole manuscript) provide valuable information and complement clinical and biochemical follow-up [5]. PROs have so far rarely been investigated in paediatric IT-IEM [6].

Implementation of meaningful PROs into research trials and clinical practice improve provider-patient communication, informed decision-making and successful medical monitoring [4, 6–8]. It is crucial to assess PROs with validated, standardised PROMs (and ObsROMs; applicable for the whole manuscript) to minimize biases. A crucial progression in this matter is the Patient-Reported Outcomes Measurement Information System (PROMIS) initiated by the U.S. National Institute of Health (NIH) [9]. This project was founded to develop, validate, and standardise PROMs that are relevant across medical conditions.

Up to date, a core set of relevant PROs completed by a list of corresponding PROMs has not been elaborated for paediatric IT-IEM patients. Such a core set, however, would allow for the comparison of self- and observer-reported data across metabolic centres and research studies as well as for a focused, targeted, economic assessment of patients’ and families’ needs.

To overcome this unmet medical need we investigated the following research questions:

1. Which PROs are relevant for paediatric IT-IEM patients, their families and specialised health care providers?
2. Which PROMs are adequate (based on predefined criteria) to measure the relevant PROs?

## Methods

### Pre-selection of potentially relevant PROs from the literature

**Fig. 1:**
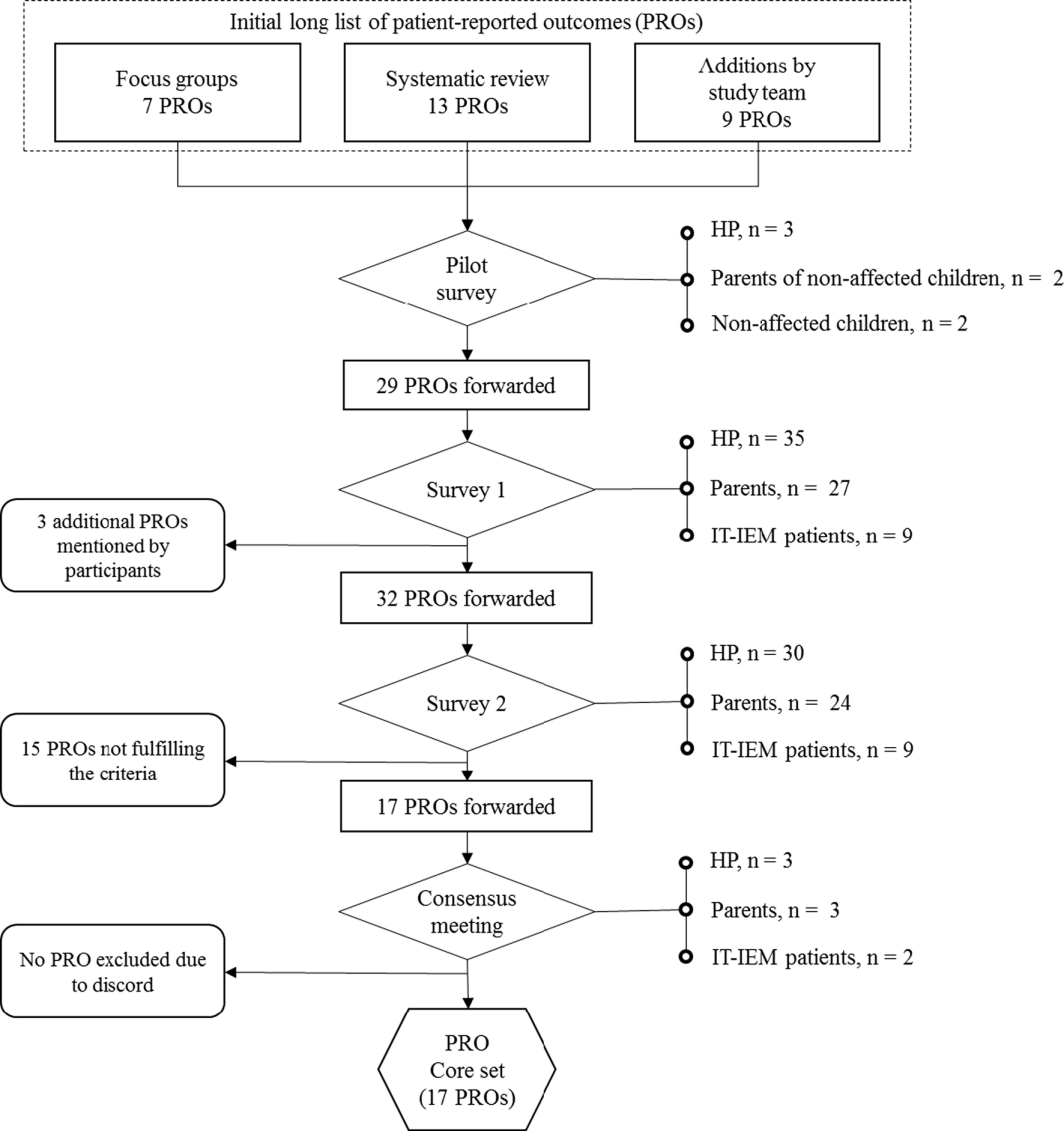
Flow chart of the modified Delphi process. In Survey two PROs were forwarded to the final consensus meeting, if they were rated as 7–9 by 70% or more of participants and as 1–3 by less than 15%. PROs were also forwarded, if they were rated as 7–9 by ≥ 90% of one stakeholder group, independently of the rating behavior of the other stakeholder group. *PRO* patient-reported outcome, *HP* health care providers, *n* sample size, *IT-IEM* intoxication-type inborn errors of metabolism

An extensive list of potentially relevant PROs in IT-IEM was assembled based on a systematic literature review [10] and qualitative focus groups [11] conducted by our research group with acute IT-IEM patients and their parents. The generated list was reviewed and completed by the authors. Overlapping and too specific PROs were excluded. For the resulting list of PROs, a survey for patients/parents and health care providers (HP) was developed. Each PRO was represented by one item (phrased in English for HP and in German for patients and parents). For each PRO-item considered “complex” by the authors, an illustrating example was given. The draft for the first online survey was piloted with a healthy sample (see Fig. 1) and adapted based on the feedback of the participants on comprehensibility, feasibility, and length.

### Recruitment process

IT-IEM patients between 12 and 18 years and parents of IT-IEM patients between 0 and 18 years treated at the University-Children’s Hospitals Zürich and Basel or at the Bregenz State Hospital were eligible. Patients and parents meeting the criteria were recruited by telephone. Health care providers (physicians, psychologists, and nutritionists) actively engaged in treating IT-IEM patients were recruited via the European Reference Network for Rare Hereditary Metabolic Disorders (metabERN; https://metab.ern-net.eu/) and the local network of the metabolic centre of the University Children’s Hospital Zürich.

### Survey procedure

The online surveys were conducted using REDCap electronic data capture tools hosted at University Children’s Hospital Zürich [12, 13]. Figure 1 provides an overview about the survey procedure. A modified Delphi process was seen as most accurate to meet the objectives of the study. In addition to a panel of specialised HP, affected patients and their parents were thereby also included in the consensus process. Two stakeholder groups were defined. One stakeholder group comprised health professionals (physicians, psychologists, nutritionist) currently working in the field of IT-IEM. The other stakeholder group comprised patients with acute and non-acute IT-IEM and parents of affected children and adolescents.

### Survey one

Participants were informed and invited by e-mail and provided a link to the survey following obtainment of informed consent. Upon request, a paper and pencil version was available. Participants were asked to complete the questionnaire within 1 month. Up to two e-mail reminders were sent in case of non-response.

Participants were asked to rate for each listed PRO how important they considered it to be measured in clinical routine and research studies in paediatric IT-IEM (nine-point Likert-scale; 1 = not at all important, 9 = very important). They were encouraged to suggest PROs not yet represented in the survey. In addition, patients and parents were asked to provide sociodemographic and disease-related data; HP were asked for information about their background such as profession and years of experience. Furthermore, every participant was asked about his or her interest to take part in the final consensus meeting.

### Survey two

Survey two was sent electronically to all respondents of survey one. All PROs from survey one were forwarded and additional PROs suggested by participants were added following revision and categorisation. For each PRO, the results of survey one were included: median score per stakeholder group, a bar chart of score-distribution by stakeholder group, and the score the participant him-/herself had given in survey one. Participants were asked to review their answers from survey one under consideration of the input by all stakeholder groups [14]. The scores of survey two on the relevance of each PRO were summarised according to the categories of the Grading of Recommendations Assessment, Development and Evaluation (GRADE) working group model [15]. PROs with a mean score of 1–3 were considered of limited importance, PROs with a median score of 4–6 as important but not critical and those with a score of 7–9 as critically important. To determine which PROs would be forwarded to the consensus meeting, the well-established rating system recommended by Williamson et al. [15–17] was applied. Criteria for inclusion were a rating as critically important by at least 70% and as of limited importance by less than 15% of participants in each stakeholder group. A PRO rated as critically important by ≥ 90% of one stakeholder group was also included and forwarded to the consensus meeting.

### Consensus meeting and qualitative interviewing

In addition to the standard Delphi approach a subsequent consensus meeting was held based on the data derived from survey two. A subsample of three HP (n = 1 physician, n = 1 psychologist, n = 1 nutritionist), three parents (mothers of n = 1 OA patient, n = 1 UCD patient, n = 1 PKU patient) and two patients (n = 1 OA patient, n = 1 PKU patient; see Fig. 1) was included. The selection of participants was content-driven (representation of all expertise fields and both acute- and non-acute diagnoses). The meeting was hosted by the two co-authors Prof. M. Huemer (physician) and F. Bösch (psychologist). Due to the ongoing COVID-pandemic the meeting was held online.

Each PRO forwarded from survey two was presented and participants of the consensus group were asked to anonymously rate them as “important, should be in the final set”, “not crucially important, should not be on the final list”, or “unsure”. Voting for each PRO was instantly visible for the participants. In case of high consensus, the group elaborated on specific characteristics of this PRO in IT-IEM and participants’ experiences with the topic. In case of discord (less than 75% of participants with the same choice), opposite opinions were explored and the voting process was repeated until a consensus was reached. All PROs rated as “important” were then included in the final list. Additionally, participants were asked for their input on corresponding PROM-questionnaires in a semi-structured way.

### Selection of corresponding PROMs

Subsequently, appropriate PROMs to measure the final set of PROs were identified based on a literature research, the expertise of the authors and the PROMIS® (Patient-Reported Outcomes Measurement Information System) database. A list of criteria for inclusion was predefined (see Additional file 1). Mandatory requirements were the availability in English, at least one community based normative sample, and the availability of both a self- and a proxy report form (for patient-oriented PROMs). Furthermore, questionnaires were reviewed for psychometric properties (validity, reliability, objectivity) and feasibility of use in paediatric IT-IEM (disease specificity, length of questionnaire, terms of use). It is noteworthy, that only questionnaires were taken into account, which reflect patients’ and parents’ subjective perception about health outcomes [3]. Standardised performance measures were precluded (e.g. test batteries to measure cognitive capacity).

## Results

### Participants, survey one and survey two

Thirty-four parents were contacted per telephone of whom 27 (79%) participated in survey one. Fourteen of their children met the age criteria and 9 (69%) agreed to participate in the study. E-mail invitations were sent to 42 health professionals of whom 35 (83%) returned survey one. Survey two was completed by 24 parents (89%), 9 patients (100%) and 30 HP (86%). 2 parents (n = 1 parent of a non-acute IT-IEM patient, 3.7%; n = 1 parent of an acute IT-IEM patient, 3.7%) and 1 HP (n = 1 physician, 2.8%) stated lack of time as the reason for their drop out. One parent of a non-acute IT-IEM patient (3.7%) and 4 HP (n = 3 physicians, 8.6%; n = 1 nutritionist, 2.8%) could not be reached until the completion of survey two. Table 1 shows the characteristics of the sample that completed survey two.

**Table 1:** Characteristics of participants in the Delphi survey two

	HP (n = 30)		Patients (n = 9)	Parents (n = 24)
*Profession (n/%)*		*Age of child (r, m)*	11–18 years (14.8)	1–19 years (9.7)
Physician	16 (53.3%)	*Gender of child (n, %)*		
Nutritionist	8 (26.7%)	Female	3 (33.3%)	12 (50%)
Psychologist	6 (20%)	Male	6 (66.7%)	12 (50%)
*Country (n/%)*		*Diagnosis*		
Switzerland	8 (26.7%)	PKU	3 (33.3%)	10 (41.7%)
Germany	7 (23.4%)	OA	2 (22.2%)	9 (37.5%)
Austria	6 (20%)	UCD	4 (44.5%)	4 (16.6%)
USA	4 (13.3%)	MSUD	0	1 (4.2%)
Italy	4 (13.3%)	*Country (n/%)*		
Netherlands	1 (3.3%)	Switzerland	7 (77.8%)	18 (75%)
*Years of experience (n/%)*		Austria	2 (22.2%)	6 (25%)
3–5 years	3 (10%)			
> 5 years	27 (90%)			

*HP* health care providers, *n* sample size, *r* range, *m* mean, *USA* United States of America, *PKU* phenylketonuria, *OA* organic acidurias, *UCD* urea cycle disorders, *MSUD* maple syrup urine disease

### PRO selection and results of the modified Delphi process

**Fig. 2:**
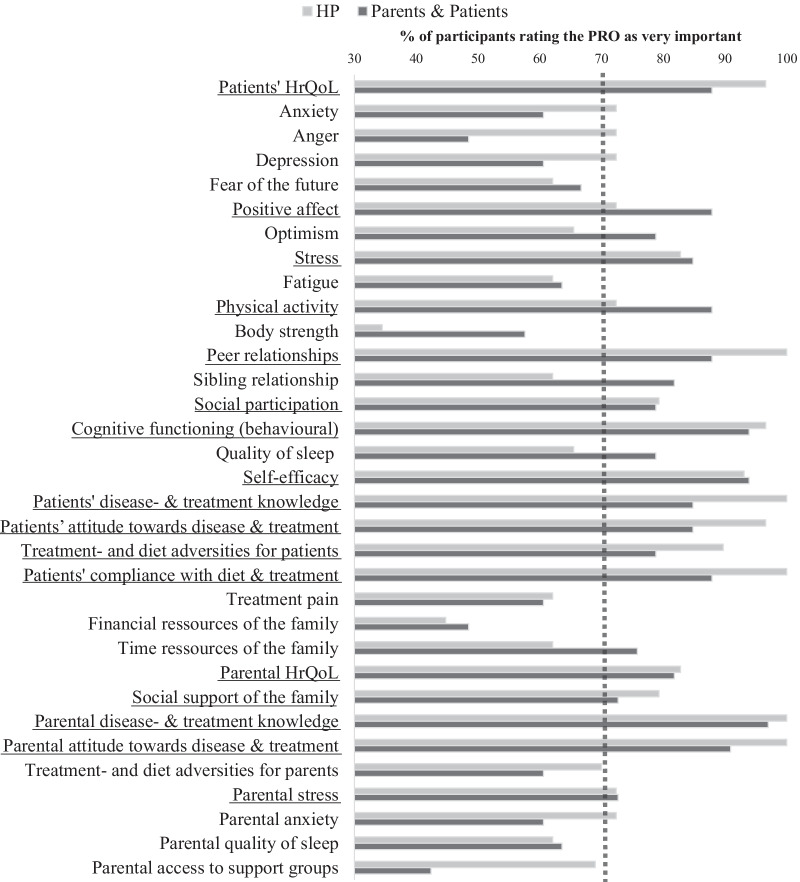
Results of survey two. The bars represent the percentage of participants rating the PRO as ‘very important’ (score 7–9) in survey 2. The dotted line marking 70% of “very important” rates represents the level of agreement for consensus to be included. Underlined PROs were included in the final core set after the final review in the consensus meeting. *HP* health care providers, *PRO* patient reported outcome, *HrQoL* health-related quality of life

Twenty-nine PROs were identified and respective items tested in a pilot sample, adapted accordingly, and consecutively presented in survey one. Three additional PROs were suggested by stakeholders (social participation, sibling relationship, and access to support groups). As described above, all 32 PROs were forwarded to survey two. Figure 2 shows the stakeholder ratings in survey two for the 32 PROs. For 22 PROs the rating of the two stakeholder groups in terms of in- or exclusion corresponded.

According to the ratings in survey two 17 PROs were forwarded to the final consensus meeting. During the consensus meeting all 17 PROs were rated as very important and eventually included in the final core set. Table 2 shows the median and interquartile range for the stakeholder ratings regarding the 17 PROs represented in the final core set. Additional file 2 provides the same parameters for all initial 32 PROs.

**Table 2:** Stakeholder ratings of the final core set of relevant PROs across the two Delphi surveys

	HP (median/IQR)		Patients and parents (median/IQR)					
	Total sample (n = 30)		Total sample (n = 33)		Non-acute sample (n = 13)		Acute sample (n = 20)	
	S1	S2	S1	S2	S1	S2	S1	S2
Patients’ HrQoL	9 (1)	9 (1)	9 (1)	9 (1)	9 (1)	9 (1)	9 (2.5)	9 (0.5)
Positive affect	7 (2)	7 (2)	8 (4)	7.5 (2)	8 (2.75)	8.5 (2)	8 (4)	8 (2.5)
Stress	7 (2)	8 (1)	8 (2.75)	8 (1)	8 (2.75)	8 (2)	8 (2)	8 (2)
Physical activity	7 (2.75)	7 (1.75)	8 (2)	8 (1.75)	8 (1.75)	8 (2)	8 (2)	8 (1.5)
Peer relationships	8 (1)	8 (1.5)	8 (2)	8 (1.5)	7 (1)	9 (1.75)	9 (1)	8 (1.5)
Social participation	–^a^	8 (1)	–^a^	8 (1)	–^a^	9 (0.75)	–^a^	8 (3)
Cognitive functioning (behavioural)	8 (2)	8 (2)	8 (2)	8.5 (2)	8.5 (2)	9 (1)	8 (2)	9 (1)
Self-efficacy (towards the disease)	8 (1)	8 (1)	9 (2)	8 (1)	9 (2.75)	8 (1.75)	8.5 (1.75)	9 (1)
Patients' disease- and treatment knowledge	8 (2)	9 (1)	9 (1)	9 (1)	9 (1)	9 (2)	9 (1.75)	9 (0)
Patients’ attitude towards disease and treatment	8 (2)	9 (1)	8.5 (2)	9 (1)	8 (1)	9 (2)	9 (2.75)	9 (1)
Treatment- and diet adversities for patients	8 (2)	8 (1)	9 (1)	8 (2)	9 (1)	9 (3.75)	8.5 (2.75)	9 (1)
Patients' compliance with diet and treatment	8 (2)	9 (1)	8.5 (2)	9 (1)	8.5 (1.75)	8 (2)	8.5 (2)	9 (1)
Parental HrQoL	7 (3)	8 (2)	8.5 (3)	8 (2)	7 (3.75)	9 (1)	9 (1.75)	7 (3.5)
Social support of the family	8 (2.75)	8 (1)	7 (4)	8 (1)	6 (2)	8 (2)	8 (3.5)	7 (2.5)
Parental disease- and treatment knowledge	9 (1)	9 (1)	9 (1)	9 (1)	8.5 (1.75)	9 (0)	9 (0.75)	9 (1)
Parental attitude towards disease and treatment	9 (1)	9 (0)	9 (1)	9 (0)	9 (2)	6.5 (0.75)	9 (1)	7 (0)
Parental stress	8 (2)	8 (2)	7.5 (3.75)	8 (2)	6.5 (2.75)	8 (2.75)	8 (7)	8 (2)

Median and IQR for stakeholder ratings (9-point Likert scale; 1 = not at all important, 9 = very important) in the two consecutive survey rounds

*HP* health care providers, *IQR* interquartile range, *n* sample size, *S1* survey one, *S2* survey two, *HrQoL* health related quality of life

^a^PRO suggested by a participant during survey one

### PROM selection

According to the predefined and prioritized list of criteria, appropriate PROMs were selected to measure the final core set of PROs. Additional file 3 shows the core list of PROMs by PRO dimension. Additional file 4 lists PROMs that were considered promising, yet could not be fully recommended based on the mandatory requirements. For two PROs of the final core set (Parental disease- and treatment knowledge, Parental attitude towards diet) no PROM fulfilled the minimal criteria for inclusion.

## Discussion

In this modified Delphi-study we aimed at defining a core outcome set of 17 PROs considered relevant by patients, parents and HP for paediatric IT-IEM and provided information on their standardised, valid assessment (PROMs). Beyond the well-established but rather broad construct of quality of life, more specific PROs like stress, physical activity, peer relationships, social participation, cognitive functioning, disease-specific self-efficacy, disease- and treatment knowledge, attitude towards, adherence to and burden of dietary treatment, family time resources, parental general health, and social support for the family were considered important. The set was developed based on the existing literature, focus groups with acute IT-IEM patients and parents and a subsequent quantitative consensus process including 64 stakeholders. The international board of specialised and experienced HP from the fields of medicine, nutrition science, and psychology represents the multidisciplinary state-of-the-art treatment approach. In the stakeholder group of patients and parents a variety of IT-IEM diagnoses is represented.

Ratings in the Delphi survey two were predominantly similar across the two stakeholder groups. This may indicate an already well-established HP-patient/parent communication. Often, a metabolic team sees patients with IT-IEM and their families for many years. This may allow for a patient-centred treatment approach with better understanding of patients’ and parents’ needs and sorrows [18].

Ratings of parents and patients with acute compared to non-acute IT-IEM, too, were similar. Both patient groups share the burden of an intoxication-type disorder and lifelong dietary treatment, and despite the absence of metabolic decompensations in e.g. PKU patients, there is previous evidence that they share most psychosocial impairments with acute-type patients [2].

Although IT-IEM are associated with heterogeneous, often burdensome physical symptoms, psychosocial PROs accounted for the majority of the final core set, suggesting that IT-IEM have an enormous impact on patients’ and families’ everyday life. A holistic treatment approach which considers not only physical symptoms but also mental and social challenges relevant to patients [19, 20] is mandatory for rare diseases like IT-IEM. In the final consensus meeting the mother of a child with MMA commented: “I don’t think it is the occasional tiredness or other ailments every now and then that bothers him/us the most. In my opinion it is much more the sorrows at a higher level that are most troublesome, the constant worrying […] is he going to have elevated temperature, how do we organise the next school trip […] that you must learn to deal with.” The inclusion of affected families in this study was crucial to guarantee the patient-centeredness and applicability of the PRO core set [21].

Psychological data are sometimes looked at sceptically by the medical system that often considers them weak and unreliable. To overcome this prejudice, we provide a list of PROMs which have been selected according to their psychometric quality following state-of-the-art scientific standards of validity, reliability, and standardisation. While the PRO set advises clinicians and researchers which parameters should be assessed, the PROM list shows how and by what means this can be achieved. The consistent application of PROMs promotes patient satisfaction and provider-patient communication and ensures comparability of research data [22]. Especially in rare diseases such as IT-IEM the collection of registry-based data and comparative analyses are crucial for the generalisability of results [6, 23], to describe the natural course, identify unmet needs and to evaluate changes induced by new treatments and interventions [3]. In this core set some well-established, broader constructs such as HrQoL are even covered by both generic (e.g. PedsQL) and disease-specific PROMs (e.g. MetabQOL/PKU-QOL). Generic PROMs compare IT-IEM patients with patients suffering from other diseases or healthy norms; disease-specific instruments have their merits in detecting changes of the addressed construct over time in a patient group [24, 25]. For more distinct, not yet widely targeted PROs such as disease- and treatment knowledge, valid instruments are not available so far. The development of missing PROMs using standardised methods is of crucial importance to take full advantage of this core set.

This study provides a first multidimensional, consensus-based core set of relevant PROs for usage in paediatric IT-IEM. A list of corresponding open access PROMs ought to facilitate the implementation in daily care and research. The underrepresentation of fathers in the consensus process is a limitation of this study, which is unfortunately rather typical for paediatric IT-IEM research [26]. Furthermore, the number of participating paediatric IT-IEM patients was relatively small and the generalisability of results is limited by the restriction to German speaking patients and parents treated at one of three metabolic centres, who share health insurance systems with high refund rates, saving them from excessive deductibles. PROs related to financial difficulties may play a more important role in other health care contexts. However, ratings of the international and experienced HP board in this study did not deviate fundamentally from parents’ and patients’ ratings. The final PRO core set should therefore be sufficiently extensive and relevant for a variety of health care systems.

Finally, there is no agreement on the optimal methodology for the development of a PRO core set. Any alternative consensus approach, such as structural interviews in vivo, may have produced a different core set. However, based on consensus finding in other rare diseases such as paediatric rheumatology [27], the online approach concerning the modified Delphi process was considered appropriate to include a larger and more diverse sample than an interview approach would have allowed.

## Conclusion

This study provides a core set of PROs relevant to paediatric IT-IEM patients, their parents and HP likewise. Corresponding PROMs are listed to ensure measurement consistency. The implementation of this core set in IT-IEM care has the potential to further advance provider-patient communication and a patient-centred treatment approach. This core set further ensures consistency in PRO assessment, both in daily care and in research. Especially in rare diseases like IT-IEM, data-comparability is of crucial importance to allow cross-national research and the inclusion of PRO-data in patient registries.

## Supplementary Information

**Additional file 1.** List of criteria for inclusion of PROMs to measure the PRO core set.

**Additional file 2.** Stakeholder ratings of all PRO across the two Delphi surveys.

**Additional file 3.** Core selection of PROMs to measure the final PRO core set.

**Additional file 4.** Additional PROMs to measure the PROs included in the core set (predefined requirements not fulfilled).

## Data Availability

Please contact the authors for data requests and/or inquiries regarding the listed PROMs.

## Abbreviations

GRADE: Grading of recommendations assessment, development and evaluation
HP: Health care providers
HrQoL: Health-related quality of life
IQR: Interquartile range
IT-IEM: Intoxication-type inborn errors of metabolism
metabERN: European reference network for hereditary metabolic diseases
MMA: Methylmalonic acidaemia
MSUD: Maple syrup urine disease
NIH: National institute of health
OA: Organic acidurias
ObsRO: Observer-reported outcomes
ObsROM: Observer-reported outcome measures
PedsQL: Pediatric quality of life inventory
PKU: Phenylketonuria
PRO: Patient-reported outcome
PROM: Patient-reported outcome measure
PROMIS: Patient-reported outcomes measurement information system
UCD: Urea cycle disorders
