# Supplementary material for: Key patient-reported outcomes in children and adolescents with intoxication-type inborn errors of metabolism: an international Delphi-based consensus
Source: Orphanet J Rare Dis. 2022 Jan 29;17:26. doi: 10.1186/s13023-022-02183-2 (PMC8800290; doi:10.1186/s13023-022-02183-2)
Supplement: Supplementary file 1 — Additional file 1. List of criteria for inclusion of PROMs to measure the PRO core set. [file 13023_2022_2183_MOESM1_ESM.docx]

**Additional file 1** – List of criteria for inclusion of PROMs to measure the PRO core set

| Dimension | Criterion | Condition | | |
| --- | --- | --- | --- | --- |
|  |  | mandatory | important | optional |
| Language | English initial form or validated English translation | 🗶 |  |  |
|  | Availability in other languages |  | 🗶 |  |
| Source of report | Self-report (PROM) | 🗶 |  |  |
|  | Proxy-report (ObsROM) | 🗶 |  |  |
| Specificity | Generic | 🗶 |  |  |
|  | Chronic generic |  | 🗶 |  |
|  | Disease-specific |  | 🗶 |  |
| Instrument lengths | Number of items < 20 |  | 🗶 |  |
| Accessibility* | Open-source (for non-commercial use) |  | 🗶 |  |
| *Objectivity* | Standardised scoring | 🗶 |  |  |
|  | Standardised interpretation | 🗶 |  |  |
| *Reliability* | Minimal internal consistency of total score: Cronbach’s α ≥ 0.6 |  | 🗶 |  |
| Reference norm | Community sample | 🗶 |  |  |
|  | Chronically ill sample |  |  | 🗶 |
|  | IT-IEM sample |  |  | 🗶 |
| Abbreviation: PROM, patient-reported outcome measure; PRO, patient-reported outcomes; ObsROM, observer-reported outcome measure; IT-IEM, Intoxication-type inborn errors of metabolism.  *Accessibility of PROM might change over time. | | | | |
