# Supplementary material for: Key patient-reported outcomes in children and adolescents with intoxication-type inborn errors of metabolism: an international Delphi-based consensus
Source: Orphanet J Rare Dis. 2022 Jan 29;17:26. doi: 10.1186/s13023-022-02183-2 (PMC8800290; doi:10.1186/s13023-022-02183-2)
Supplement: Supplementary file 2 — Additional file 2. Stakeholder ratings of all PRO across the two Delphi surveys. [file 13023_2022_2183_MOESM2_ESM.docx]

**Additional file 2** – Stakeholder ratings of all PRO across the two Delphi surveys

|  | HP (Median/IQR) | |  | Patients & Parents (Median/IQR) | | | | | |
| --- | --- | --- | --- | --- | --- | --- | --- | --- | --- |
|  | Total sample | |  | Total sample | | Non-acute sample | | Acute sample | |
|  | S1 | S2 |  | S1 | S2 | S1 | S2 | S1 | S2 |
| Patients’ HrQoL | 9 (1) | 9 (1) |  | 9 (1) | 9 (1) | 9 (1) | 9 (1) | 9 (2.5) | 9 (0.5) |
| Anxiety | 7.5 (2) | 7 (2) |  | 7 (4) | 7 (2) | 7.5 (2.75) | 7 (3) | 6.5 (5.5) | 7 (1.5) |
| Anger | 8 (1.75) | 7 (3) |  | 7 (4) | 6.5 (3) | 7 (3.75) | 5 (3.75) | 5 (6.75) | 8 (3.5) |
| Depression | 7 (2) | 7 (2) |  | 7 (4) | 7 (2) | 7.5 (3) | 8 (5) | 5 (7) | 8 (1.5) |
| Fear of the future | 8 (2) | 7 (2) |  | 7 (5) | 7 (2) | 6.5 (4.5) | 8 (4.75) | 7.5 (5.75) | 7 (2) |
| Positive affect | 7 (2) | 7 (2) |  | 8 (4) | 7.5 (2) | 8 (2.75) | 8.5 (2) | 8 (4) | 8 (2.5) |
| Optimism | 7 (3) | 7 (2.5) |  | 8 (2) | 7 (2.5) | 7.5 (2) | 7.5 (1.75) | 8 (3.5) | 7 (2) |
| Stress | 7 (2) | 8 (1) |  | 8 (2.75) | 8 (1) | 8 (2.75) | 8 (2) | 8 (2) | 8 (2) |
| Fatigue | 7 (3) | 7 (2) |  | 8 (2) | 7 (2) | 7.5 (2) | 8 (3.75) | 8 (5.25) | 8 (2) |
| Physical activity | 7 (2.75) | 7 (1.75) |  | 8 (2) | 8 (1.75) | 8 (1.75) | 8 (2) | 8 (2) | 8 (1.5) |
| Body strength | 6 (2) | 6 (2) |  | 7 (2.75) | 6 (2) | 7 (1) | 7.5 (4) | 7.5 (3) | 7 (2.5) |
| Peer relationships | 8 (1) | 8 (1.5) |  | 8 (2) | 8 (1.5) | 7 (1) | 9 (1.75) | 9 (1) | 8 (1.5) |
| Sibling relationship^a^ | -- | 7 (2) |  | -- | 8 (2) | -- | 9 (1.75) | - | 8 (2) |
| Social participation^a^ | -- | 8 (1) |  | -- | 8 (1) | -- | 9 (0.75) | - | 8 (3) |
| Cognitive functioning (behavioural) | 8 (2) | 8 (2) |  | 8 (2) | 8.5 (2) | 8.5 (2) | 9 (1) | 8 (2) | 9 (1) |
| Quality of sleep | 6 (3) | 7 (2) |  | 8 (2) | 8 (2) | 7.5 (1.75) | 8 (1.75) | 8 (2) | 8 (2) |
| Self-efficacy (towards the disease) | 8 (1) | 8 (1) |  | 9 (2) | 8 (1) | 9 (2.75) | 8 (1.75) | 8.5 (1.75) | 9 (1) |
| Patients' disease- & treatment knowledge | 8 (2) | 9 (1) |  | 9 (1) | 9 (1) | 9 (1) | 9 (2) | 9 (1.75) | 9 (0) |
| Patients’ attitude towards disease & treatment | 8 (2) | 9 (1) |  | 8.5 (2) | 9 (1) | 8 (1) | 9 (2) | 9 (2.75) | 9 (1) |
| Treatment- and diet adversities for patients | 8 (2) | 8 (1) |  | 9 (1) | 8 (2) | 9 (1) | 9 (3.75) | 8.5 (2.75) | 9 (1) |
| Patients' compliance with diet & treatment | 8 (2) | 9 (1) |  | 8.5 (2) | 9 (1) | 8.5 (1.75) | 8 (2) | 8.5 (2) | 9 (1) |
| Treatment pain | 7 (2.75) | 7 (1) |  | 7 (4) | 7 (1) | 7 (2.75) | 7 (6) | 8 (4.75) | 7 (3) |
| Financial resources of the family | 6.5 (3) | 6 (1.5) |  | 5 (4) | 6 (1.5) | 5 (4) | 6.5 (5.25) | 6.5 (5.5) | 6 (3) |
| Time resources of the family | 7 (2) | 7 (2) |  | 8 (4) | 7.5 (1.5) | 5.5 (3) | 9 (1) | 9 (1.75) | 7 (3) |
| Parental HrQoL | 7 (3) | 8 (2) |  | 8.5 (3) | 8 (2) | 7 (3.75) | 9 (1) | 9 (1.75) | 7 (3.5) |
| Social support of the family | 8 (2.75) | 8 (1) |  | 7 (4) | 8 (1) | 6 (2) | 8 (2) | 8 (3.5) | 7 (2.5) |
| Parental disease- & treatment knowledge | 9 (1) | 9 (1) |  | 9 (1) | 9 (1) | 8.5 (1.75) | 9 (0) | 9 (0.75) | 9 (1) |
| Parental attitude towards disease & treatment | 9 (1) | 9 (0) |  | 9 (1) | 9 (0) | 9 (2) | 6.5 (0.75) | 9 (1) | 7 (0) |
| Treatment- and diet adversities for parents | 7 (2) | 7 (2) |  | 7 (4.75) | 7 (2) | 7 (3) | 9 (7.5) | 7.5 (7.75) | 9 (1.5) |
| Parental stress | 8 (2) | 8 (2) |  | 7.5 (3.75) | 8 (2) | 6.5 (2.75) | 8 (2.75) | 8 (7) | 8 (2) |
| Parental anxiety | 6.5 (3) | 8 (2) |  | 7.5 (4) | 7.5 (2) | 5.5 (4) | 7.5 (1.75) | 8 (6.5) | 7 (3.5) |
| Parental quality of sleep | 6 (3) | 7 (2) |  | 7.7 (5) | 7 (2) | 6.5 (3.75) | 8 (3.5) | 7.5 (6.25) | 5 (2.5) |
| Parental access to support groups^a^ | -- | 7 (2) |  | -- | 7 (2) | -- | 7 (5.5) | -- | 6 (2.5) |

Median and IQR for stakeholder ratings (9-point Likert scale; 1 = not at all important, 9 = very important) in the two consecutive survey rounds.
Abbreviation: HP, health care providers; IQR, interquartile range; S1, first survey round; S2, second survey round; HrQoL, health-related quality of life.

^a^ PRO suggested by a participant during the first survey round.
