# Supplementary material for: Key patient-reported outcomes in children and adolescents with intoxication-type inborn errors of metabolism: an international Delphi-based consensus
Source: Orphanet J Rare Dis. 2022 Jan 29;17:26. doi: 10.1186/s13023-022-02183-2 (PMC8800290; doi:10.1186/s13023-022-02183-2)
Supplement: Supplementary file 3 — Additional file 3. Core selection of PROMs to measure the final PRO core set. [file 13023_2022_2183_MOESM3_ESM.docx]

**Additional file 3** – Core selection of PROMs to measure the final PRO core set

| PROM | Specificity | Sub-scales | Source | Age-range (in years) | Item  count | Existing norms | Tested  IT-IEM-populations | Availability in English | Open access^a,b^ |
| --- | --- | --- | --- | --- | --- | --- | --- | --- | --- |
| **Patients’ health-related quality of life (HrQoL)** | | | | | | | | | |
| Pediatric quality of life inventory (PedsQL)^1^ | generic | Physical, emotional, social and school functioning | Sr | 5-18 | 23 | Community norm^2^:  USA (2007); n = 9,500 children; age range = 5 – 18.1 years | PKU^3–7^;  OA &  UCD^5^ | ✓  & >60 full or partial translations | ✓ |
|  |  |  | Pr | 2-4 | 21 |  |  |  |  |
|  |  |  |  | 5-18 | 23 |  |  |  |  |
| PROMIS Pediatric Global Health – fixed form 7^8^ | generic | Global factor, no sub-scales | Sr | 8-17 | 7 | Community norm^9^:  USA (2018); n = 3,973 children; age range = 8 – 17 years | 🗶 | ✓^c^ | ✓ |
|  |  |  | Pr | 5-17 | 7 | Community norm^9^:  USA (2018); n = 5,206 parents; age range of children = 5 – 17 years | 🗶 |  |  |
| KIDSCREEN-27^10^ | chronic generic | Physical, psychological, autonomy & parent relations, peers & social support, school environment | Sr | 8-18 | 27 | European Community norm^10^: AT, CZ, FR, DE, GR, HU, IRL, PL, ES, SWE, CH, NL, GBR (2003-2005); n ≈ 21,277 children; age range = 8 – 18 years | PKU & OA^11,12^ | ✓  &  >40 full or partial translations | ✓ |
|  |  |  | Pr | 8-18 | 27 | European Community norm^10^: AT, CZ, FR, DE, GR, HU, IRL, PL, ES, SWE, CH, NL, GBR (2003-2005); n ≈ 15,748 parents; age range children = 8 – 18 years  Community norm^13^: DE (2009-2012); n = 1,658 parents; age range children = 8 – 18 years |  |  |  |
| MetabQOL^14^ | Disease specific | Physical, mental, social, disease severity | Sr | 4-7 | 10 | 🗶 | OA & UCD^14^ | ✓  &  DE, TUR | ✓ |
|  |  |  |  | 8-18 | 28 |  |  |  |  |
|  |  |  | Pr | 4-7 | 10 |  |  |  |  |
|  |  |  |  | 8-18 | 28 |  |  |  |  |
| PKU-QOL^15^ | Disease specific | Physical, mental, social; Additional: disease severity | Sr | 9-11 | 10 | PKU sample^7^: FR, DE, ITA, NL, ES, TUR, GBR (2011-2012); n = 306 children; age range = 8 – 17 years | PKU^7^ | ✓  &  DE, ES, FR, ITA, NL | 🗶 |
|  |  |  |  | 12-17 | 28 |  |  |  |  |
|  |  |  | Pr | 0-17 | 10 | PKU sample^7^: FR, DE, ITA, NL, ES, TUR, GBR (2011-2012); n = 253 parents; age range children = 8 – 17 years |  |  |  |
|  |  |  |  |  | 28 |  |  |  |  |
| **Patient’s positive affect** | | | | | | | | | |
| PROMIS positive affect – fixed form 8^16^ | generic | Global factor | Sr | 8-17 | 8 | Community norm^9^:  USA (2018); n = 3,973 children; age range = 8 – 17 years | 🗶 | ✓^c^ | ✓ |
|  |  |  | Pr | 5-17 | 8 | Community norm^9^:  USA (2018); n = 5,206 parents; age range of children = 5 – 17 years |  |  |  |
| **Patient’s perceived stress** | | | | | | | | | |
| PROMIS psychological stress experience – fixed form 8^17^ | generic | Global factor | Sr | 8-17 | 8 | Community norm^9^:  USA (2018); n = 3,973 children; age range = 8 – 17 years | 🗶 | ✓^c^ | ✓ |
|  |  |  | Pr | 5-17 | 8 | Community norm^9^:  USA (2018); n = 5,206 parents; age range of children = 5 – 17 years |  |  |  |
| **Patients’ physical activity** | | | | | | | | | |
| PROMIS physical activity – fixed form 8^18^ | generic | Global factor | Sr | 8-17 | 8 | Community norm^9^:  USA (2018); n = 3,973 children; age range = 8 – 17 years | 🗶 | ✓^c^ | ✓ |
|  |  |  | Pr | 5-17 | 8 | Community norm^9^:  USA (2018); n = 5,206 parents; age range of children = 5 – 17 years |  |  |  |
| **Patients’ peer relationships** | | | | | | | | | |
| PROMIS peer relationships – fixed form 8^19^ | generic | Global factor | Sr | 8-17 | 8 | Community norm^9^:  USA (2018); n = 3,973 children; age range = 8 – 17 years | 🗶 | ✓^c^ | ✓ |
|  |  |  | Pr | 5-17 | 8 | Community norm^9^:  USA (2018); n = 5,206 parents; age range of children = 5 – 17 years |  |  |  |
| **Patients’ social participation** | | | | | | | | | |
| n/a | | | | | | | | | |
| **Patients’ cognitive functioning (behavioural)** | | | | | | | | | |
| PROMIS cognitive functioning – fixed form 7^20^ | generic | Global factor | Sr | 8-17 | 7 | Community norm^20^:  USA (2011); n = 1,409 parents; age range of children = 7 – 17 years  Cancer sample^21^:  USA (2014); n = 515 patients; age range = 7 – 17 years | 🗶 | ✓^c^ | ✓ |
|  |  |  | Pr | 8-17 | 7 | Community norm^20^:  USA (2011); n = 1,409 parents; age range of children = 7 – 17 years  Cancer sample^21^:  USA (2014); n = 515 parents; age range of children = 7 – 17 years |  |  |  |
| **Patients’ self-efficacy** | | | | | | | | | |
| NIH Toolbox self efficacy – fixed form^22^ | generic | Global factor | Sr | 8-12 | 10 | Community norm^23^:  USA (2020); n = 1,120 children; age range = 8 – 12 years | 🗶 | ✓ | ✓ |
|  |  |  | Pr | 13-17 | 10 | Community norm^23^:  USA (2020); n = 1,128 parents; age range of children = 13 – 17 years |  |  |  |
| **Patients disease- and treatment knowledge** | | | | | | | | | |
| n/a (see supplementary table) | | | | | | | | | |
| **Patients’ attitude towards their disease (and treatment)** | | | | | | | | | |
| n/a (see supplementary table) | | | | | | | | | |
| **Treatment- and diet adversities for patients** | | | | | | | | | |
| n/a (see supplementary table) | | | | | | | | | |
| **Patient’s compliance with diet & treatment** | | | | | | | | | |
| n/a (see supplementary table) | | | | | | | | | |
| **Parental health-related quality of life (HrQoL)** | | | | | | | | | |
| Short form health questionnaire (SF-36)^24^ | generic | Global factor & physical functioning, physical role, pain, general health, vitality, social function, emotional role, mental health | Sr  (par) | 16+ | 36 | Short selection:  Community norm^25^: GBR (1993); n = 13,042 adults; age range = 18-64 years  Chronic and non-chronic condition norm^26^: USA (1989); n = 9,385 adults; age range = 18 - 103 years | PKU^7,27^ | ✓  &  >60 full or partial translations | ✓ |
| PROMIS Scale v1.2 – Global Health^28^ | generic | Global factor &  mental health, physical health | Sr  (par) | 18+ | 10 | Community norm^29^: USA (2005-2008); n = 21,133 adults; age range = 18-65+ years  Community norm^30^: USA (2010); n = 4,184 adults; age range = 18-65+ years | 🗶 | ✓^c^ | ✓ |
| **Social support of the family** | | | | | | | | | |
| PROMIS – Emotional support^31^ | generic | Global factor | Sr  (par) | 18+ | 8 | 🗶 | 🗶 | ✓^c^ | ✓ |
| Oslo 3 social support scale (OSSS-3)^32^ | generic | Global factor | Sr  (par)  (child) | 12+ | 3 | Community sample^33^: EU (2008); n = 15,945 children and adolescents; age range = 12 – 18 years  Community sample^34^: EU (2006); n = 14,387 children and adolescents; age range = 12 – 18 years  Community sample^35^: IRL (2011); n = 10,364 adults; age range = 18 – 65+ years | 🗶 | ✓ | ✓ |
| **Parental disease- and treatment knowledge** | | | | | | | | | |
| n/a | | | | | | | | | |
| **Parental attitude towards their child’s disease (and treatment)** | | | | | | | | | |
| n/a | | | | | | | | | |
| **Parental stress** | | | | | | | | | |
| Perceived Stress Scale (PSS-10)^36^ | generic | Global factor | Sr  (par) | 18+ | 10 | Community norm^37^: USA (1983-2009); n = 2,332 participants; age range = 18 – 65+ years Community norm^38^: DE (2014); n = 2,527 participants; age range = 14 – 95 years | PKU^39^ | ✓ | ✓ |
| Impact on family scale^40^ | chronic generic | Global factor & financial impact; familial-social impact, personal strain, mastery | Sr  (par) | 18+ | 27 | Chronic condition sample^41^: USA (1984); n = 219 families; age range children: 0 – 11 years;  Chronic condition sample^42^: USA (1994); n = 352 families; age range children: 5-8 years  Chronic condition sample^43^: DE (2014); n = 219 families; age range children: 0-18 years | 🗶 | ✓ | ✓ |
| Abbreviation: PROM, Patient-reported outcome measure; PRO, Patient-reported outcome; IT-IEM, intoxication-type inborn errors of metabolism; Sr, self-report; Pr, proxy-report; USA, United States of America; n, sample size; PKU, Phenylketonuria; OA, Organic acidurias; UCD, Urea cycle disorders; PROMIS, Patient Reported Outcome Measurement Information System; AT, Austria; CZ, Czech Republic; FR, France; DE, Germany; GR, Greece; HU, Hungary; IRL, Ireland; PL, Poland; ES, Spain; SWE, Sweden; CH, Switzerland; NL, Netherland; GBR, Great Britain; TUR, Turkey; ITA, Italy; n/a, not available; par, parents; EU, European Union.  ^a^Commercial research not included; accessibility of PROM might change over time.  ^b^PROMIS instruments and corresponding manuals can be found online ([www.healthmeasures.net](http://www.healthmeasures.net)); for inquiries regarding the other listed instruments you can contact F. Bösch or M. Huemer for more information.  ^c^Translations of PROMIS instruments can be found online ([www.healthmeasures.net](http://www.healthmeasures.net)). New translations are constantly added and are based on a standardised translation process (forward and back-translation, multiple expert reviews, harmonization across languages, and cognitive debriefing). | | | | | | | | | |

**References**

1. Varni JW, Seid M, Kurtin PS. PedsQL^TM^ 4.0: Reliability and Validity of the Pediatric Quality of Life Inventory^TM^ Version 4.0 Generic Core Scales in Healthy and Patient Populations. *Med Care*. 2001;39(8):800-812. doi:10.1097/00005650-200108000-00006

2. Varni JW, Limbers CA, Burwinkle TM. Impaired health-related quality of life in children and adolescents with chronic conditions: A comparative analysis of 10 disease clusters and 33 disease categories/severities utilizing the PedsQL^TM^ 4.0 Generic Core Scales. *Health Qual Life Outcomes*. 2007;5. doi:10.1186/1477-7525-5-43

3. Cazzorla C, Cegolon L, Burlina AP, et al. Quality of Life (QoL) assessment in a cohort of patients with Phenylketonuria. *BMC Public Health*. 2014;14(1):1243. doi:10.1186/1471-2458-14-1243

4. Demirdas S, Maurice-Stam H, Boelen CCA, et al. Evaluation of quality of life in PKU before and after introducing tetrahydrobiopterin (BH4); a prospective multi-center cohort study. *Mol Genet Metab*. 2013;110(SUPPL.):S49-S56. doi:10.1016/j.ymgme.2013.09.015

5. Jamiolkowski D, Kölker S, Glahn EM, et al. Behavioural and emotional problems, intellectual impairment and health-related quality of life in patients with organic acidurias and urea cycle disorders. *J Inherit Metab Dis*. 2016;39(2):231-241. doi:10.1007/s10545-015-9887-8

6. Vieira Neto E, Maia Filho HS, Monteiro CB, et al. Quality of life and adherence to treatment in early-treated Brazilian phenylketonuria pediatric patients. *Brazilian J Med Biol Res*. 2018;51(2):1-10. doi:10.1590/1414-431x20176709

7. Bosch AM, Burlina A, Cunningham A, et al. Assessment of the impact of phenylketonuria and its treatment on quality of life of patients and parents from seven European countries. *Orphanet J Rare Dis*. 2015;10(1):80. doi:10.1186/s13023-015-0294-x

8. Forrest CB, Bevans KB, Pratiwadi R, et al. Development of the PROMIS® pediatric global health (PGH-7) measure. *Qual Life Res*. 2014;23(4):1221-1231. doi:10.1007/s11136-013-0581-8

9. Carle AC, Bevans KB, Tucker CA, Forrest CB. Using nationally representative percentiles to interpret PROMIS pediatric measures. *Qual Life Res*. 2020;1:3. doi:10.1007/s11136-020-02700-5

10. Ravens-Sieberer U, Auquier P, Erhart M, et al. The KIDSCREEN-27 quality of life measure for children and adolescents: Psychometric results from a cross-cultural survey in 13 European countries. *Qual Life Res*. 2007;16(8):1347-1356. doi:10.1007/s11136-007-9240-2

11. Thimm E, Schmidt LE, Heldt K, Spiekerkoetter U. Health-related quality of life in children and adolescents with phenylketonuria: unimpaired HRQoL in patients but feared school failure in parents. *J Inherit Metab Dis*. 2013;36(5):767-772. doi:10.1007/s10545-012-9566-y

12. Eminoglu TF, Soysal SA, Tumer L, Okur I, Hasanoglu A. Quality of life in children treated with restrictive diet for inherited metabolic disease. *Pediatr Int*. 2013;55(4):428-433. doi:10.1111/ped.12089

13. Barkmann C, Otto C, Meyrose AK, et al. Psychometrics and Norms of the Quality of Life Inventory KIDSCREEN in Germany. *Diagnostica*. 2021;67(1):2-12. doi:10.1026/0012-1924/a000257

14. Zeltner NA, Baumgartner MR, Bondarenko A, et al. Development and psychometric evaluation of the metabQoL 1.0: A quality of life questionnaire for paediatric patients with intoxication-type inborn errors of metabolism. In: *JIMD Reports*. Vol 37. ; 2016:27-35. doi:10.1007/8904_2017_11

15. Regnault A, Burlina A, Cunningham A, et al. Development and psychometric validation of measures to assess the impact of phenylketonuria and its dietary treatment on patients’ and parents’ quality of life: The phenylketonuria - Quality of life (PKU-QOL) questionnaires. *Orphanet J Rare Dis*. 2015;10(1). doi:10.1186/s13023-015-0261-6

16. Forrest CB, Ravens-Sieberer U, Devine J, et al. Development and Evaluation of the PROMIS® Pediatric Positive Affect Item Bank, Child-Report and Parent-Proxy Editions. *J Happiness Stud*. 2018;19(3):699-718. doi:10.1007/s10902-016-9843-9

17. Bevans KB, Gardner W, Pajer K, Riley AW, Forrest CB. Qualitative development of the PROMIS® pediatric stress response item banks. *J Pediatr Psychol*. 2013;38(2):173-191. doi:10.1093/jpepsy/jss107

18. Tucker CA, Bevans KB, Becker BD, Teneralli R, Forrest CB. Development of the PROMIS pediatric physical activity item banks. *Phys Ther*. 2020;100(8):1393-1410. doi:10.1093/ptj/pzaa074

19. DeWalt DA, Thissen D, Stucky BD, et al. PROMIS pediatric peer relationships scale: Development of a peer relationships item bank as part of social health measurement. *Heal Psychol*. 2013;32(10):1093-1103. doi:10.1037/a0032670

20. Lai J-S, Butt Z, Zelko F, et al. Development of a Parent-Report Cognitive Function Item Bank Using Item Response Theory and Exploration of its Clinical Utility in Computerized Adaptive Testing. *J Pediatr Psychol*. 2011;36(7):766-779. doi:10.1093/jpepsy/jsr005

21. Lai JS, Zelko F, Krull KR, et al. Parent-reported cognition of children with cancer and its potential clinical usefulness. *Qual Life Res*. 2014;23(4):1049-1058. doi:10.1007/s11136-013-0548-9

22. Salsman JM, Schalet BD, Merluzzi T V., et al. Calibration and initial validation of a general self-efficacy item bank and short form for the NIH PROMIS®. *Qual Life Res*. 2019;28(9):2513-2523. doi:10.1007/s11136-019-02198-6

23. Paolillo EW, McKenna BS, Nowinski CJ, Thomas ML, Malcarne VL, Heaton RK. NIH Toolbox® Emotion Batteries for Children: Factor-Based Composites and Norms. *Assessment*. 2020;27(3):607-620. doi:10.1177/1073191118766396

24. Ware JE, Sherbourne CD. The MOS 36-item short-form health survey (Sf-36): I. conceptual framework and item selection. *Med Care*. 1992;30(6):473-483. https://www.jstor.org/stable/3765916?seq=1#metadata_info_tab_contents. Accessed July 14, 2020.

25. Jenkinson C, Coulter A, Wright L. Short form 36 (SF 36) health survey questionnaire: Normative data for adults of working age. *Br Med J*. 1993;306(6890):1437-1440. doi:10.1136/bmj.306.6890.1437

26. Stewart AL. Functional Status and Well-being of Patients With Chronic Conditions. *JAMA*. 1989;262(7):907. doi:10.1001/jama.1989.03430070055030

27. Palermo L, MacDonald A, Limback E, et al. Emotional health in early-treated adults with phenylketonuria (PKU): Relationship with cognitive abilities and blood phenylalanine. *J Clin Exp Neuropsychol*. 2020;42(2):142-159. doi:10.1080/13803395.2019.1696753

28. Hays RD, Bjorner JB, Revicki DA, Spritzer KL, Cella D. Development of physical and mental health summary scores from the patient-reported outcomes measurement information system (PROMIS) global items. *Qual Life Res*. 2009;18(7):873-880. doi:10.1007/s11136-009-9496-9

29. Stone A, Choi S, Lai J-S, et al. The Patient-Reported Outcomes Measurement Information System (PROMIS) developed and tested its first wave of adult self-reported health outcome item banks: 2005–2008. *J Clin Epidemiol*. 2010;63(11):1179-1194. doi:10.1016/j.jclinepi.2010.04.011

30. Barile JP, Reeve BB, Smith AW, et al. Monitoring population health for Healthy People 2020: Evaluation of the NIH PROMIS® Global Health, CDC Healthy Days, and satisfaction with life instruments. *Qual Life Res*. 2013;22(6):1201-1211. doi:10.1007/s11136-012-0246-z

31. Cyranowski JM, Zill N, Bode R, et al. Assessing social support, Companionship, And distress: National institute of health (NIH) toolbox adult social relationship scales. *Heal Psychol*. 2013;32(3):293-301. doi:10.1037/a0028586

32. Kocalevent RD, Berg L, Beutel ME, et al. Social support in the general population: Standardization of the Oslo social support scale (OSSS-3). *BMC Psychol*. 2018;6(1):1-8. doi:10.1186/s40359-018-0249-9

33. Ravens-Sieberer U, Erhart M, Gosch A, Wille N. Mental health of children and adolescents in 12 European countries—results from the European KIDSCREEN study. *Clin Psychol Psychother*. 2008;15(3):154-163. doi:10.1002/cpp.574

34. Dalgard OS, Dowrick C, Lehtinen V, et al. Negative life events, social support and gender difference in depression. *Soc Psychiatry Psychiatr Epidemiol*. 2006;41(6):444-451. doi:10.1007/s00127-006-0051-5

35. Van Lente E, Barry MM, Molcho M, et al. Measuring population mental health and social well-being. *Int J Public Health*. 2012;57(2):421-430. doi:10.1007/s00038-011-0317-x

36. Cohen S, Kamarck T, Mermelstein R. A global measure of perceived stress. *J Health Soc Behav*. 1983:385-396.

37. Cohen S, Janicki-Deverts D. Who’s Stressed? Distributions of Psychological Stress in the United States in Probability Samples from 1983, 2006, and 2009. *J Appl Soc Psychol*. 2012;42(6):1320-1334. doi:10.1111/j.1559-1816.2012.00900.x

38. Klein EM, Brähler E, Dreier M, et al. The German version of the Perceived Stress Scale - psychometric characteristics in a representative German community sample. *BMC Psychiatry*. 2016;16(1). doi:10.1186/s12888-016-0875-9

39. Irannejad F, Dehghan M, Mehdipour Rabori R. Stress and quality of life in parents of children with phenylketonuria. *J Child Adolesc Psychiatr Nurs*. 2018;31(2-3):48-52. doi:10.1111/jcap.12207

40. Stein REK, Riessman CK. The development of an impact-on-family scale: Preliminary findings. *Med Care*. 1980;18(4):465-472. doi:10.1097/00005650-198004000-00010

41. Stein REK, Jessop DJ. Does Pediatric Home Care Make a Difference for Children with Chronic Illness? Findings from the Pediatric Ambulatory Care Treatment Study. *Pediatrics*. 1984;73(6).

42. Silver EJ, Stein REK, Dadds MR. Moderating effects of family structure on the relationship between physical and mental health in urban children with chronic illness. *J Pediatr Psychol*. 1996;21(1):43-56. doi:10.1093/jpepsy/21.1.43

43. Dehn LB, Korn-Merker E, Pfäfflin M, Ravens-Sieberer U, May TW. The Impact on Family Scale: Psychometric analysis of long and short forms in parents of children with epilepsy. *Epilepsy Behav*. 2014;32:21-26. doi:10.1016/j.yebeh.2013.12.030
