# Supplementary material for: Key patient-reported outcomes in children and adolescents with intoxication-type inborn errors of metabolism: an international Delphi-based consensus
Source: Orphanet J Rare Dis. 2022 Jan 29;17:26. doi: 10.1186/s13023-022-02183-2 (PMC8800290; doi:10.1186/s13023-022-02183-2)
Supplement: Supplementary file 4 — Additional file 4. Additional PROMs to measure the PROs included in the core set (predefined requirements not fulfilled). [file 13023_2022_2183_MOESM4_ESM.docx]

**Additional file 4** – Additional PROMs to measure the PROs included in the core set (predefined requirements not fulfilled)

| PROM | Specificity | Sub-scales | Source | Age-range (in years) | Item count | Existing norms | Tested  IT-IEM-populations | Availability in English | Open access^a^ |
| --- | --- | --- | --- | --- | --- | --- | --- | --- | --- |
| **Patient’s perceived stress** | | | | | | | | | |
| Perceived Stress Scale (PSS-10)^1^ | generic | Global factor | Sr  (par) | 14+ | 10 | Community norm^2^: USA (1983-2009); n = 2,332 participants; age range = 18 – 65+ years Community norm^3^: DE (2014); n = 2,527 participants; age range = 14 – 95 years | 🗶 | ✓^a^ | ✓ |
| **Patients’ cognitive functioning (behavioural)** | | | | | | | | | |
| Behavior Rating Inventory of Executive Function (BRIEF)^4^ | generic | Inhibit, shift, emotional control, initiate, working memory, plan/organize, organization of materials, monitor | Sr | 11-18 years | 80 | 🗶 | 🗶 | ✓ | 🗶 |
|  |  |  | Pr | 5-18 years | 86 | Community norm^4^:  USA (2018); n = 1,419 parents; age range of children = 5 – 17 years |  |  |  |
| **Patients disease- and treatment knowledge** | | | | | | | | | |
| Disease specific questionnaire for IT-IEM^5^ | disease specific | Total score | Sr | 7+ | 5-8  & 1 o.q. | 🗶 | PKU  & OA  & UCD^5^ | 🗶 & DE, NL | ✓ |
| **Patients’ attitude towards their disease (and treatment)** | | | | | | | | | |
| Illness perception questionnaire revised  (IPQ-R)^6^ | chronic generic  /  disease specific^b^ | Identity, consequences, personal control, timeline, treatment control, coherence, emotional representation, psychological attributions, possible causes | Sr | Not yet validated for children | 38  & 14 (symptoms)  &  18 (causes) | 🗶 | 🗶 | ✓  &  >15 full or partial translations | ✓ |
| **Treatment- and diet adversities for patients** | | | | | | | | | |
| MetabQOL^7^ | disease specific | Sub-scale:  diet | Sr | 4-18 | 2 | 🗶 | OA & UCD^7^ | ✓  &  DE, TUR | ✓ |
|  |  |  | Pr | 4-18 | 2 |  |  |  |  |
| MetabQOL^7^ | disease specific | Sub-scale: medication | Sr | 4-18 | 2 (+1 if feeding tube) | 🗶 | OA & UCD^7^ | ✓  &  DE, TUR | ✓ |
|  |  |  | Pr | 4-18 | 2 (+1 if feeding tube) |  |  |  |  |
| PKU-QOL^8^ | disease specific | Sub-scale: PKU diet and medical formula | Sr | 9-11 | 17 | PKU sample^9^: FR, DE, ITA, NL, ES, TUR, GBR (2011-2012); n = 306 children; age range = 8 – 17 years | PKU^8^ | ✓  &  DE, ES, FR, ITA, NL | 🗶 |
|  |  |  |  | 12-17 | 22 |  |  |  |  |
|  |  |  | Pr | 0-17 | 16 | PKU sample^9^: FR, DE, ITA, NL, ES, TUR, GBR (2011-2012); n = 253 parents; age range children= 8 – 17 years |  |  |  |
| **Patient’s compliance with diet & treatment** | | | | | | | | | |
| Self-Care-Inventory-revised  (SCI-R – adapted) | disease specific | Total score | Sr | 7-18 | 10 | 🗶 | 🗶 | 🗶 DE | ✓ |
| Abbreviation: IT-IEM, intoxication-type inborn errors of metabolism; Sr, self-report; par, parents; USA, United States of America; n, sample size; DE, Germany; Pr, proxy-report; o.q., open question; PKU, Phenylketonuria; OA, Organic acidurias; UCD, Urea cycle disorders; NL, Netherland; TUR, Turkey; FR, France; ITA, Italy; ES, Spain; GBR, Great Britain. ^a^Commercial research not included; accessibility of PROM might change over time; for inquiries regarding the listed instruments you can contact F. Bösch or M. Huemer. ^b^Several disease-specific versions for a number of common illnesses. More versions are being added regularly on <https://ipq.h.uib.no/html/citing.html>. | | | | | | | | | |

**References**

1. Cohen S, Kamarck T, Mermelstein R. A global measure of perceived stress. *J Health Soc Behav*. 1983:385-396.

2. Cohen S, Janicki-Deverts D. Who’s Stressed? Distributions of Psychological Stress in the United States in Probability Samples from 1983, 2006, and 2009. *J Appl Soc Psychol*. 2012;42(6):1320-1334. doi:10.1111/j.1559-1816.2012.00900.x

3. Klein EM, Brähler E, Dreier M, et al. The German version of the Perceived Stress Scale - psychometric characteristics in a representative German community sample. *BMC Psychiatry*. 2016;16(1). doi:10.1186/s12888-016-0875-9

4. Gioia GA, Isquith PK, Guy SC, Kenworthy L, Baron IS. Behavior rating inventory of executive function. *Child Neuropsychol*. 2000;6(3):235-238. doi:10.1076/chin.6.3.235.3152

5. Zeltner NA, Welsink-Karssies MM, Landolt MA, et al. Reducing complexity: Explaining inborn errors of metabolism and their treatment to children and adolescents. *Orphanet J Rare Dis*. 2019;14(1):248. doi:10.1186/s13023-019-1236-9

6. Moss-Morris R, Weinman J, Petrie K, Horne R, Cameron L, Buick D. The revised Illness Perception Questionnaire (IPQ-R). *Psychol Heal*. 2002;17(1):1-16. doi:10.1080/08870440290001494

7. Zeltner NA, Baumgartner MR, Bondarenko A, et al. Development and psychometric evaluation of the metabQoL 1.0: A quality of life questionnaire for paediatric patients with intoxication-type inborn errors of metabolism. In: *JIMD Reports*. Vol 37. ; 2016:27-35. doi:10.1007/8904_2017_11

8. Regnault A, Burlina A, Cunningham A, et al. Development and psychometric validation of measures to assess the impact of phenylketonuria and its dietary treatment on patients’ and parents’ quality of life: The phenylketonuria - Quality of life (PKU-QOL) questionnaires. *Orphanet J Rare Dis*. 2015;10(1):1-18. doi:10.1186/s13023-015-0261-6

9. Bosch AM, Burlina A, Cunningham A, et al. Assessment of the impact of phenylketonuria and its treatment on quality of life of patients and parents from seven European countries. *Orphanet J Rare Dis*. 2015;10(1):80. doi:10.1186/s13023-015-0294-x
